# An Innovative Bioengineering Approach to Investigate the Response of Melanin-Rich Cells to Intense Pulsed Light (IPL)

**DOI:** 10.3390/cells15100859

**Published:** 2026-05-08

**Authors:** Kirsty Goncalves, Kous Shah, Victoria Maltman, Yuwen Chen, Nicole Barrett, Georgia Abraham, Ilaria Ambrogio, Teresa DiColandrea, John Snowball, Stefan Przyborski

**Affiliations:** 1 Department of Biosciences, Durham University, Durham DH1 3LE, UK; kirsty.e.goncalves@durham.ac.uk (K.G.); victoria.b.maltman@durham.ac.uk (V.M.); nicole.barrett@durham.ac.uk (N.B.); georgia.abraham@durham.ac.uk (G.A.); 2 Procter & Gamble, Reading RG2 0QE, UKchen.y.46@pg.com (Y.C.); ambrogio.i@pg.com (I.A.); snowball.jm@pg.com (J.S.); 3 Procter & Gamble, Cincinnati, OH 45201, USA; dicoland@hotmail.com; 4 ReproCELL Europe Ltd., Glasgow G20 0XA, UK

**Keywords:** in vitro, human bioengineered skin tissue, heat shock, Intense Pulsed Light, apoptosis, melanin, thermolysis

## Abstract

**Highlights:**

**What are the main findings?**

- Development of an in vitro approach to better understand the underlying molecular and cellular events that govern the response to Intense Pulsed Light (IPL).
- Innovative application of a bioengineering approach to model the response of melanin-rich cells to IPL treatments.

**What are the implications of the main findings?**

- The in vitro platform is capable of more in-depth and higher throughput screening than current approaches.
- Industrial and academic applications can provide further insight into the cellular and molecular impacts of light treatments at a physiological level.

**Abstract:**

Light-based therapies are becoming increasingly = more mainstream, not only within the medical science space, but also within the fields of cosmetic dermatology and personal grooming. Intense Pulsed Light (IPL) harnesses the ability of the natural chromophore–melanin to absorb light energy, which is translated into heat energy and consequently results in targeted thermolysis of cells rich in melanin. This mechanistic pathway lends itself to a wide range of applications, including long-term hair removal, skin rejuvenation, the treatment of unwanted pigmentation, and the treatment of ophthalmic conditions. The development of home use devices (HUDs) for the delivery of IPL-mediated hair removal has facilitated the self-administration of photothermal treatments and reduced reliance on clinical settings. In this study, we demonstrate a pioneering approach to model aspects of IPL-induced thermal induction and selective thermolysis in a complex human skin tissue equivalent. Our approach utilised a deactivated HUD with disabled safety features that allowed for the exposure of tissue constructs to high-fluence IPL. We demonstrate an increase in biomarkers consistent with increased cellular temperature, induction of apoptosis, and increased pro-inflammatory cytokine release following extreme treatment regimens, all of which correlate with an increased fluence and/or increased number of IPL pulses delivered. This method allowed for the identification of cellular events evoked by increasing fluence and extreme-exposure regimes.

## 1. Introduction

Intense Pulsed Light (IPL) therapy is a technique that utilises broad-spectrum visible light to selectively target chromophores within skin and other tissues [1]. Dermatologically, the principal target of IPL is melanin, the most abundant chromophore in skin and hair, and major determinant of skin tone and hair colour [2]. IPL energy is absorbed by melanin, which raises the temperature of melanin-rich cells, leading to thermal shock and selective thermolysis. This basic mechanism leads to targeted cellular destruction and enables a wide range of applications relating to cosmetic and dermatological practises [3]. For example, the ability to target pigment-rich cells in the dermis has been utilised in the treatment of birth marks [4]. Similarly, the targeting of melanin-rich cells in the epidermis is frequently used as a means to reduce the appearance of age spots and unwanted age-associated pigmentary changes whilst promoting an even skin tone [5,6,7,8,9]. When the primary target are populations of melanin-rich cells located in the bulb region of the hair follicle, IPL is also routinely used as a method of semi-permanent hair removal [10].

The popularity of IPL as an epilatory treatment has experienced significant growth recently due to the emergence of home use devices (HUDs), which have allowed for treatments to transition from the beauty salon into consumer homes. This methodology harnesses the ability of melanin within the bulb region of the follicle to absorb the energy of a wavelength within the range emitted by the IPL device (often 400–1200 nm) [11]. Light energy is absorbed by melanin-rich cells within the hair follicle bulb, which, in turn, is converted into heat energy that damages the surrounding tissue at the base of the follicle and suppresses hair regrowth [12]. Targeted cells include melanocytes and keratinocytes within the matrix of the bulb region located above the dermal papilla that differentiate into areas of the hair shaft and inner root sheath. The effectiveness of IPL as a hair removal practice relies on contrast in energy absorption between the cells of the follicle and the surrounding skin cells, which achieves specificity. For example, IPL treatments are more effective in individuals with lighter skin tones and dark hairs, whilst potentially having alternative outcomes in consumers of dark skin tones due to the prevalence of unwanted side effects [13].

Current approaches used to study the specific cellular and molecular pathways that govern IPL’s biological activity rely heavily on clinical trials [14,15,16,17]. However, clinical trials are associated with an array of limitations, including that they are both expensive and time-consuming whilst also requiring an invasive tissue sample collection for downstream in-depth analysis, which is often hindered by reliance upon specialist staff, ethical approval, and volunteer engagement. They are, however, considered an industry standard, as they involve the study of skin and hair in situ and involve interactions between the complex cell types within the native tissue. Similarly, the most widely adopted laboratory-based approaches assess the impact of IPL on single follicles or ex vivo skin/hair explants, which again are restricted due to sample availability and are often associated with a limited cell viability and lifespan in vitro [3,18].

For this reason, the ability to model the impact of IPL on a suitable biological system mimicking the function of native skin in vitro would represent a novel and significant advancement within the field. A limited number of studies exist that examine the impact of IPL on cellular systems in vitro, but these lack complexity, involving conventional two-dimensional (2D) cultures of cells in either mono- or co-culture [19,20,21]. In such studies, IPL is delivered within the cultureware and in the presence of cell culture medium, meaning that there is an increased distance between the target cells and the IPL device. This could impact the light-scattering dynamics of the system and output of the study. Structurally, the complexity of a 2D culture is far removed from the multi-cellular, stratified, dynamic nature of native skin tissues; therefore, its predictive capacity and ability to model nuanced biological processes are equally simplified.

We have previously reported the development of a bioengineered pigmented human skin equivalent (PHSE) and its response to photomodulation and hypopigmentary agents alongside its ability to model photodamage in vitro [22,23]. In this study, we present a novel application for this bioengineered tissue construct: modelling the biological effect of IPL on a melanin-rich complex tissue. The PHSE we have developed relies on the endogenous deposition of extracellular matrix (ECM) from resident human dermal fibroblasts that promotes basement membrane formation de novo and provides paracrine and cellular support to the overlying epidermis consisting of both keratinocytes and melanocytes. We have demonstrated that the lack of animal derived products and enhanced cellular communication between the three cell types are essential factors for the production and distribution of melanin throughout the tissue, particularly the formation of melanin supranuclear caps consistent with native skin [23,24,25].

Although our PHSE lacks the presence of a hair follicle, it contains melanin-rich cells: both melanocytes that synthesise melanin and keratinocytes rich in melanin due to enhanced melanin transfer exhibited in our system. Therefore, in this study, we detail the innovative use of our PHSE to elucidate the molecular events that occur in melanin-rich cell types (melanocytes and keratinocytes following IPL-exposure within a complex more physiologically relevant tissue environment. We utilise an adapted HUD with a deactivated safety system capable of delivery high-fluence repeated treatments to provide fundamental insights into the cellular and molecular response to extreme IPL-exposures. This platform could provide a tool to better understand the impact of IPL at a cellular level, including the up- or downregulation of cellular processes in response to IPL exposure. We present an innovative approach utilising our PHSE as a cellular tool, capable of modelling IPL-induced photothermolysis in vitro, thereby providing a platform capable of both fundamental biological insights and efficacy assessments.

## 2. Materials and Methods

### 2.1. Pigmented Human Skin Equivalent (PHSE) Engineering

Commercially available primary cells were used to create PHSEs, including human neonatal keratinocytes (HEKn, ThermoFisher Scientific, Loughborough, UK), darkly pigmented human neonatal epidermal melanocytes (HEMn-DP, ThermoFisher Scientific), and neonatal human dermal fibroblasts (HDFn, ThermoFisher Scientific). Cells were maintained in culture flasks up to 80% confluence prior to seeding in tissue constructs.

PHSEs were generated as previously described [22,23,26]. Briefly, a dermal compartment was engineered through culture of HDFn within Alvetex^®^ Scaffold (ReproCELL Europe Ltd., Glasgow, UK) for 19 days in submerged culture to promote endogenous production of extracellular matrix (ECM). HEKn and HEMn-DP were seeded onto mature dermal compartments at a ratio of 1:10 (melanocytes:keratinocytes) in submerged culture to promote cellular proliferation. After 48 h, cultures were raised to the air–liquid interface (ALI) to promote epidermal stratification and further maintained for 21 days prior to use in experiments.

### 2.2. Intense Pulsed Light (IPL) Exposure

PHSEs were unclipped from their culture inserts and placed within a plastic 90 mm Petri dish (ThermoFisher Scientific) atop the L50(300)A-IPL energy sensor (Ophir^®^ Photonics, Darmstadt, Germany). The Braun Silk Expert Pro 5 home use device (HUD) (PL5124, Procter & Gamble, Reading, UK) was used to deliver consumer-relevant IPL energy to the surface of the PHSE. In-line with the genuine consumer experience, the factory pre-set calibration was put in place throughout the experimental period, and subsequent in-house calibration was not required. Control PHSEs were also unclipped from their culture inserts and the HUD was passed across them without emitting an energy pulse to act as a sham control in order to determine that the biological effects are a consequence of IPL exposure directly and not the manipulation/handling of the PHSE. A custom-made deactivation jig was fitted to the head of the HUD to bypass the skin contact, safety mechanism, and allow for pulse delivery in vitro. The Vega laser power and energy meter (Ophir^®^ Photonics, Darmstadt, Germany) was used to detect and record fluence in each experimental condition. Treatment regimens represent low (3.24 Jcm^−2^), medium (5.22 Jcm^−2^), and high (6.07 Jcm^−2^) device settings, and PHSEs were either exposed to a single-pulse treatment, multiple subsequent pulses delivered immediately after one another, or chronic exposure whereby a single pulse was delivered twice weekly for a period of two weeks.

### 2.3. Skin Tone Measurements

Objective measurements of skin tone, including melanin index (MI), were obtained as previously described [22,23] through the removal of PHSEs from their culture inserts and use of Skin ColourCatch (Delfin Technologies, Surrey, UK).

### 2.4. Cleaved Caspase 3 ELISA

Samples of culture medium were harvested from PHSEs 48 h after IPL treatment. The Human Cleaved Caspase-3 ELISA kit (Abcam, Cambridge, UK, ab220655) was used as per the manufacturer’s instructions in order to quantify cleaved caspase 3 activity. The assay was measured using a BioTek ELx800 microplate reader at 405 nm.

### 2.5. MTT Cell Viability Assay

MTT assay was used to identify areas of cell death within IPL-exposed tissue constructs. PHSEs were washed in PBS and incubated with 1 mgmL^−1^ MTT solution consisting of thiazolyl blue tetrazolium bromide (Sigma-Aldrich, Dorset, UK) dissolved in phenol red free Dulbecco’s modified eagle’s medium (ThermoFisher Scientific) and incubated at 37 °C for 20 min. PHSEs were then imaged using the Dino-Lite Edge AM7915MZT digital microscope (Absolute Services Ltd., Hertfordshire, UK) and lysed in acidified isopropanol for 20 min at room temperature. Absorbance of the resultant solution was measured at 570 nm using a BioTek ELx800 microplate reader (Agilent, Santa Clara, CA, USA).

### 2.6. Cytokine Multiplex Analysis

Samples of culture medium were harvested from PHSEs 48 h after IPL treatment. Analysis of the cytokine content of the medium was performed by Eve Technologies (Calgary, AB, Canada) and the Human Cytokine Proinflammatory Focussed 15-Plex Discovery^®^ Assay Array (HDF15) was performed.

### 2.7. Paraffin Wax Embedding

Tissue constructs were processed and embedded as previously described [22]. Samples were fixed in 4% paraformaldehyde (Sigma-Aldrich) overnight at 4 °C and then dehydrated through a series of ethanols. Samples were then incubated in Histoclear (SLS, Nottingham, UK) alone, mixed 1:1 with molten paraffin wax (ThermoFisher Scientific) and then molten paraffin wax alone. Samples were embedded in plastic moulds (CellPath, Newton, UK) in paraffin wax and sectioned transversely using a microtome (Leica RM2125RT). Then 5 μm sections were placed on charged microscope slides (ThermoFisher Scientific).

### 2.8. Haematoxylin and Eosin (H&E) Staining

Samples were deparaffinised in Histoclear and rehydrated through a series of ethanol baths. Samples were stained in Mayer’s haematoxylin (Sigma-Aldrich) for 5 min, followed by alkaline ethanol for 30 s to blue the nuclei. Slides were then dehydrated in ethanol and incubated with eosin (Sigma-Aldrich) for 30 s before being further dehydrated. Finally, slides were cleared in Histoclear and mounted with coverslips using Omnimount mountant (SLS, Scientific Laboratory Supplies, London, UK).

### 2.9. Fontana–Masson (FM) Staining

Fontana–Masson (FM) melanin staining was achieved using a commercially available kit (Abcam, ab150669), and the manufacturer’s instructions were followed.

### 2.10. Immunofluorescence

Sections were deparaffinised in Histoclear and rehydrated through a series of ethanols. Antigen retrieval was performed at 95 °C in citrate buffer pH 6 (Sigma-Aldrich) for 20 min. Blocking and permeabilization followed for 1 h at room temperature in a solution consisting of 20% neonatal calf serum (NCS, Sigma-Aldrich) and 0.4% Triton X-100 (Sigma-Aldrich) in phosphate-buffered saline (PBS). Samples were then incubated with the primary antibody (Table S1) diluted in blocking/permeabilization buffer overnight at 4 °C, followed by three washes in PBS. Slides were then incubated with the appropriate secondary antibody (donkey anti-rabbit Alexa Flour 488 or donkey anti-mouse Alexa Flour 594, ThermoFisher Scientific, 1:1000) and the nuclear dye Hoechst (ThermoFisher Scientific, 1:10,000) for 1 h at room temperature. Samples were then washed three times in PBS and mounted using Vectashield Hardset mountant (Vector Laboratories, Peterborough, UK), prior to imaging.

### 2.11. Epidermal Whole-Mount Staining

In order to visualise a large area of the stratum basale, the epidermal layer was separated from the underlying dermis through enzymatic digestion, as previously described [22,23]. Unclipped PHSEs were incubated in dispase (Sigma-Aldrich) for 20 min at room temperature, and the epidermis was peeled from the dermal compartment with forceps. Following this, the epidermis was peeled from the tissue construct using forceps and washed in PBS. The epidermal samples were then either immunostained as described above or underwent analysis using the TUNEL assay.

### 2.12. TUNEL Assay

The DeadEnd™ Fluorometric TUNEL system (Promega, Southampton, UK) was used to detect cellular apoptosis. The manufacturer’s instructions were followed. Briefly, isolated epidermal samples were permeabilised in 0.2% Triton X-100 for 5 min prior to equilibration. Samples were then incubated at 37 °C for 60 min in a reaction mixture consisting of nucleotide mix, rTdT enzyme, and equilibration buffer. The reaction was then stopped through the addition of 2X SSC solution for 15 min prior to washing in PBS with the addition of Hoechst for 5 min. Epidermal whole-mounts were then mounted using Vectashield mounting medium (Vecta Labs, Oldbury, UK).

### 2.13. Microscopy

H&E and FM images were captured using the Leica ICC50 high-definition camera and brightfield microscope (Leica, London, UK). Immunofluorescence and TUNEL images were taken using the Zeiss 880 confocal microscope using the Airyscan and Zen (3.11) software (Carl Zeiss Ltd., Cambridge, UK).

### 2.14. Biometric Quantification

Epidermal thickness measurements were obtained using ImageJ (1.54s) software as previously described [26,27]. Briefly, the straight-line tool was used to trace the distance from the stratum basale to the stratum corneum, including only the viable epidermal layers.

The TUNEL assay was quantified using the multipoint tool in ImageJ to count the number of TUNEL-positive nuclei (stained) and the total number of nuclei (Hoechst stained), which was then expressed as a percentage. Similarly, melanocyte density was calculated by also using the multipoint tool to count the number of TRP1-positive cells in the field of view and were expressed as a function of area (cells per mm^2^), as previously described [23].

### 2.15. Statistical Analysis

GraphPad Prism (10.6.1) software was used to measure the statistical significance by use of a one-way ANOVA with Tukey’s post hoc test. Significance is depicted graphically * = *p* < 0.05, ** = *p* < 0.01, *** = *p* < 0.001, **** = *p* < 0.0001. In all cases, 3 independent experiments were conducted (referred to as n), with a minimum of 3 technical measurements taken within each independent experiment. Graphical data are shown as mean ± SEM.

## 3. Results

### 3.1. Delivery of Intense Pulsed Light (IPL) In Vitro

**Figure 1:**
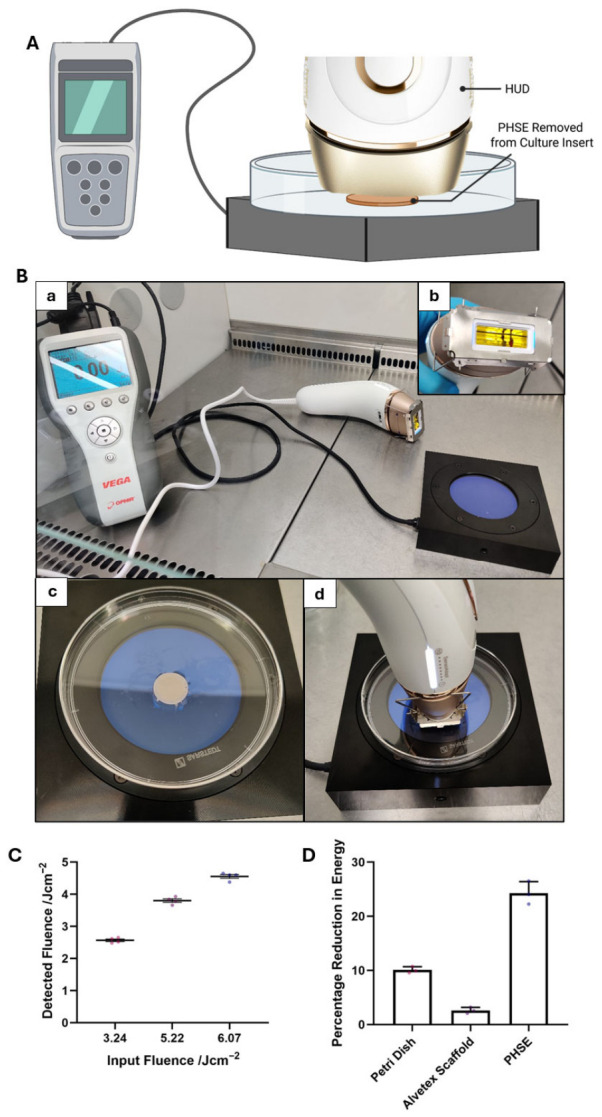
Delivery of Intense Pulsed Light (IPL) energy in vitro. Schematic representation of IPL delivery from a home use device (HUD) in vitro (**A**). The pigmented human skin equivalent (PHSE) is placed within a standard Petri dish on top of an energy sensor connected to a power meter. Photographic representation of IPL delivery apparatus in vitro, including HUD, power meter and energy sensor (**Ba**). To allow for IPL pulse delivery in vitro, a bespoke de-activation jig was fitted to the head of the IPL device, which bypasses the skin surface detection safety system (**Bb**). Photographic representation of PHSE in situ prior to IPL delivery (**Bc**) and HUD in contact with PHSE during pulse emission (**Bd**). Relationship between input fluence emitted directly from HUD and fluence detected with PHSE in place (**C**) (data represent mean ± SEM, n = 4, three measurements were taken from four independently engineered PHSE constructs). Percentage of energy reduced by each element of the in vitro methodology, including: Petri dish, Alvetex^®^ Scaffold and PHSE (**D**) (data represent mean ± SEM, n = 3, three measurements were taken per condition along with the mean of three independently constructed PHSEs).

The Braun Silk Expert Pro 5 HUD was used to deliver IPL energy to the surface of PHSEs (Figure 1A). The unclipped PHSE (Figure 1(Bc)) was irradiated atop of Petri dish and an energy meter with connecting sensor (Figure 1(Ba)) with an IPL pulse delivered at 90° to the construct (Figure 1(Bd)). The contact between the PHSE and HUD is an important aspect of the methodology, not only as this reflects genuine consumer treatment, but also to reduce uncontrolled light scattering and ensure the PHSE is exposed to the complete fluence. In order to achieve this in vitro, a custom-made deactivation jig (Figure 1(Bb)) was fitted to the head of the HUD to allow for pulse emission without the need for complete skin contact.

With the PHSE in situ, the fluence detected from beneath the tissue construct, was slightly reduced compared with the fluence measured directly from the HUD (Figure 1C). In order to investigate which components of the apparatus were responsible for this reduction in detectable energy, the percentage energy reduced by each component of the apparatus was measured (Figure 1D). This indicated that ~10% of energy was reduced by the presence of the Petri dish, ~2.5% reduced by the addition of Alvetex^®^ Scaffold to the Petri dish, and a combined reduction of ~25% with the addition of the cell-containing complete tissue construct (PHSE) to the Petri dish. This reduction in energy is most likely a combination of light scattering, absorption, and partial reflection at interfaces by the tissue construct and equipment. For example, the significant reduction in detectable energy when the Petri dish is in situ is likely particularly impacted by partial reflectance, which, given that there are two interfaces in the light path, this could contribute to energy loss and as the refractive index of the material remains unknown, we were not able to quantify this phenomenon.

However, as the HUD is placed directly in contact with the PHSE, the tissue construct receives the complete input fluence with little environmental scattering. This represents consumer-relevant IPL-delivery to the human bioengineered tissue and models the in vivo experience, allowing for a reliable investigation into the cellular responses evoked by IPL exposure.

### 3.2. Cellular Temperature Increases with IPL Treatment

**Figure 2:**
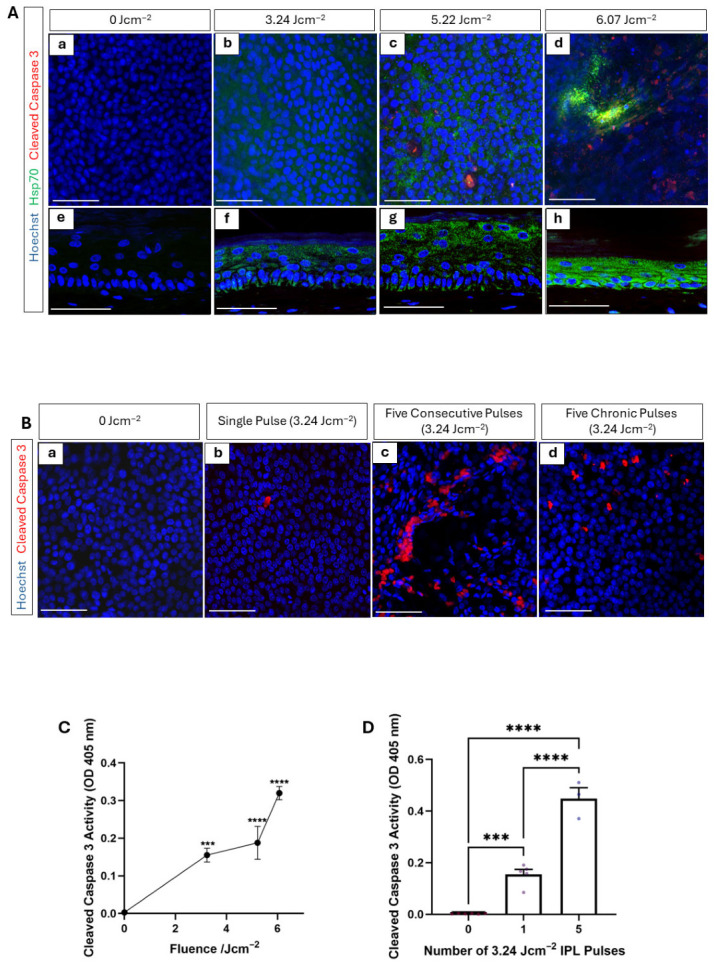
IPL exposure increases cellular temperature in a fluence-dependent manner. Immunodetection of biomarkers consistent with raised cellular temperature, including hsp70 (green, expressed 38–41 °C) and cleaved caspase 3 (red, expressed >41 °C) with nuclei stained in blue by Hoechst (**A**). PHSEs were exposed to IPL and harvested for analysis 48 h post-exposure. Representative immunofluorescence images of whole-mount epidermis, depicting an overview of the stratum basale (**Aa**–**d**) and transverse cross-sections of PHSEs demonstrating increased biomarker expression when exposed to increasing IPL fluence (**Ae**–**h**). PHSEs were either subjected to a single low fluence IPL pulse (**Bb**), five consecutive low-fluence IPL pulses (**Bc**), or five chronic low–fluence pulses delivered every 2–3 days across a 2 week period (**Bd**) or not exposed to IPL (**Ba**) and harvested for analysis 48 h post-final exposure. Micrographs illustrating whole-mount epidermal immunodetection of cleaved caspase 3 (red, nuclei stained blue) across the stratum basale, with increasing staining evident with increased number of low-fluence IPL pulses (**B**). Cleaved caspase 3 activity was quantified from culture medium 48 h post-IPL exposure through ELISA analysis. Cleaved caspase 3 activity significantly increased with increasing fluence (**C**) and number of low-fluence IPL pulses (**D**) (data represent mean ± SEM, n = 3, representative of three measurements taken from three independently constructed PHSEs). Scale Bars: 50 μm, *** = *p* < 0.001, **** = *p* < 0.0001.

PHSEs were exposed to low (3.24 Jcm^−2^), moderate (5.22 Jcm^−2^), and high (6.07 Jcm^−2^) fluence, representative of varied levels of HUD intensities, and maintained in culture for a further 48 h post-treatment to allow for time for protein-level changes to be observable. The biomarkers hsp70 and cleaved caspase 3 are known to be expressed by tissues that undergo heat shock at temperatures within the 38–41 °C range and in excess of 41 °C, respectively [28,29,30,31,32,33]. Expression of these markers was elevated across the stratum basale with increasing fluence, as demonstrated by whole-mount epidermal staining (Figure 2(Aa–d)). No positive staining was detectable for either hsp70 or cleaved caspase 3 in the stratum basale of PHSEs that were not exposed to IPL (Figure 2(Aa)). Expression of hsp70 emerged at low fluence (Figure 2(Ab)) and increased uniformly at moderate fluence (Figure 2(Ac)). Cleaved caspase 3, however, was not detectable at low fluence, with some areas of positive staining identifiable at moderate fluence and the number of stained areas increased further at high fluence. This coincided with a reduction in the area of hsp70 staining, but an increase in staining intensity at high fluence (Figure 2(Ad)).

A cross-sectional view of the tissue confirms these observations, as hsp70 expression can be seen to increase in the epidermis in a dose-dependent manner with increasing fluence (Figure 2(Ae–h)). Together, these data infer indirectly, in the absence of direct intracellular temperature measurements, that high-fluence treatments raised cellular temperature as expected. This suggests that the tissue construct responded in the expected manner, in-line with the biological mechanism of IPL, through temperature elevation.

Furthermore, repeated low-fluence treatments of either a single pulse, five consecutive pulses delivered in a single treatment, or five chronic pulses delivered every 2–3 days over a two-week period reveal increased expression of cleaved caspase 3 (Figure 2B). Similarly to the delivery of a single pulse, PHSEs were returned to culture following treatment and harvested 48 h after their final IPL treatment. Whole-mount staining demonstrates cleaved caspase 3 expression focussed specifically within the stratum basale, the epidermal layer most rich in melanin, and expression increased with multiple IPL treatments, with the most staining visible after five consecutive treatments. The cleaving of caspase 3 also represents the commitment step in the apoptotic cascade, therefore acts as a biomarker capable of detecting cells that reached the threshold temperature required to undergo apoptosis [34]. This suggests that, in this case, more cells have reached the minimum threshold for apoptosis in PHSEs exposed to multiple consecutive IPL pulses rather than a single pulse. The increased expression is likely due to the cumulative impact of multiple heating and cooling events contributing to the observed tissue dysfunction.

Quantification of cleaved caspase 3 activity through ELISA of culture medium taken from PHSEs 48 h post-treatment also further supports this finding, as it demonstrates a statistically significant fluence-dependent increase (Figure 2C), with even low-fluence treatment resulting in a significant increase in cleaved caspase 3 activity compared with untreated PHSEs. Similarly, a single low-fluence treatment resulted in a significant increase in cleaved caspase 3 activity, whereas five consecutive low-fluence treatments resulted in an increase in cleaved caspase 3 activity that was both statistically significant from the control and single-pulse samples (Figure 2D).

Together, these data evidence cellular changes in PHSEs consistent with expected raised temperature, indicating that the tissue model is responsive to IPL. As the tissue construct is rich in melanin, this is expected and supports the notion that melanin within the system has absorbed light energy from the IPL emission, leading to raised cellular temperature. To better understand the cellular consequence of this, signs of apoptosis within the PHSEs exposed to IPL were identified.

### 3.3. IPL-Induced Selective Thermolysis of Melanin-Rich Cells

**Figure 3:**
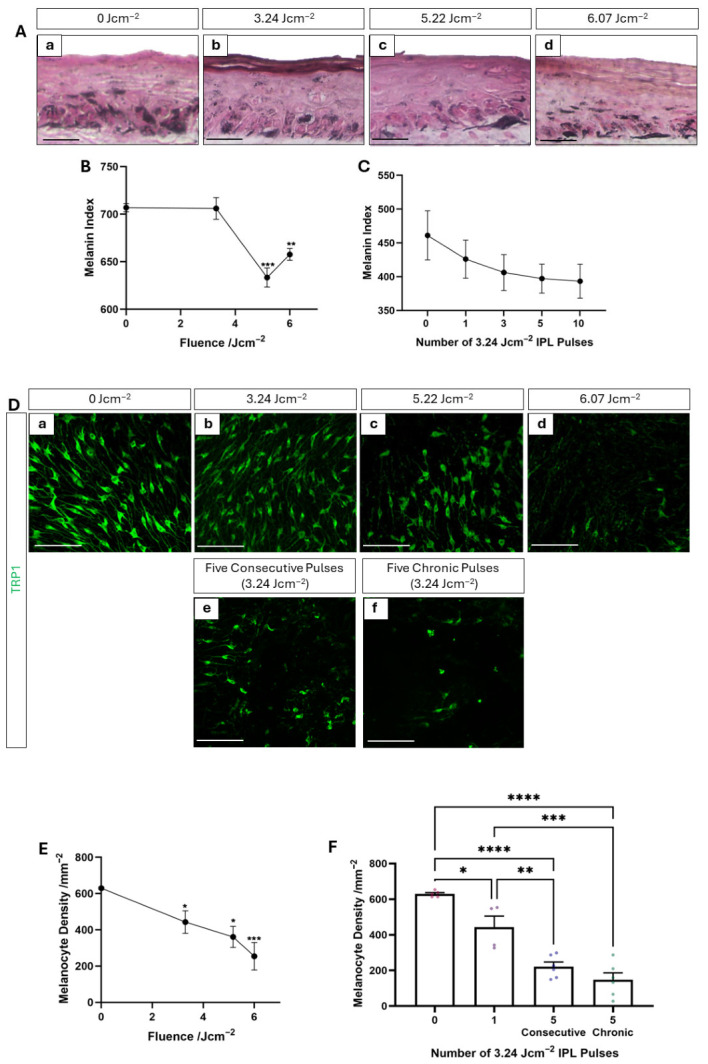
Reduction in melanin-rich cells post-IPL exposure is indicative of selective destruction. Fontana–Masson (FM) staining reveals melanin content (black) of PHSEs exposed to 0–6.07 Jcm^−2^ IPL and harvested 48 h following exposure (**Aa**–**d**). A reduction in melanin distribution throughout the epidermis can be observed up to moderate (5.22 Jcm^−2^) fluence. Quantification of melanin index evidences a reduction in overall pigmentation of the tissue with increasing fluence (**B**) and increasing number of consecutive low-fluence pulses (**C**) (data represent mean ± SEM, n = 3 representative of three measurements taken from three independently constructed PHSEs). Epidermal whole-mount immunofluorescence staining of the melanocyte marker TRP1 (green) illustrates melanocyte distribution across the stratum basale (**Da**–**f**). Melanocyte density decreases with increasing fluence and five pulses delivered both consecutively and chronically across a 2 week period, every 2–3 days, with PHSEs harvested 48 h after final IPL treatment. Quantification of melanocyte density confirms this observation with statistically significant reductions that correlate both with increasing fluence (**E**) and the number of pulses delivered consecutively or chronically (**F**) (data represent mean ± SEM, n = 3, representative of five images taken from three technical samples obtained from three independently constructed PHSEs). Scale Bars: 50 μm, * = *p* < 0.05, ** = *p* < 0.01, *** = *p* < 0.001, **** = *p* < 0.0001.

Both desired and unwanted changes to complexion and skin pigmentation are associated with high-fluence salon-only IPL treatments in vivo as a consequence of selectively targeting melanin-rich cells [13]. Due to the safety mechanisms that have been disabled for this study, these effects were not experienced following consumer HUD usage. However, in vitro, changes in pigmentation of the tissue construct infer information regarding the impact of the treatment to the intended cellular target: melanin-rich cells. Fontana–Masson staining highlights melanin content of tissue cross-sections, with melanocytes appearing as dendritic cells located in the stratum basale containing high levels of melanin. Melanin can also be observed as deposits throughout the epidermis, generally located apical to the nucleus within keratinocytes (Figure 3A). However, the frequency of melanin deposits appears to decrease with increasing fluence with the PHSE exposed to moderate fluence, containing only a small number of melanin deposits within the epidermal compartment.

This finding is confirmed by melanin index measurements, which demonstrate a significant reduction in melanin index at 5.22 Jcm^−2^ with a slight increase at 6.07 Jcm^−2^, but still significantly reduced compared with control samples (Figure 3B). A similar trend is observed when the number of consecutive low-fluence pulses is increased and a pulse-dependent reduction in melanin index is observed (Figure 3C). A decrease in melanin supports the notion of targeted destruction of melanin containing cells and indirectly substantiates the concept of IPL-induced cell death at low-to-moderate fluence, whereas high-fluence treatment likely impacts melanocyte function as is associated with a increase in melanin content compared with low–moderate treatment conditions.

To more directly demonstrate the effects of IPL on cells with high melanin content, we focused specifically on melanocytes, as these are the most melanin-abundant cell type in the epidermis. Whole-mount epidermal analysis of TRP1, a melanocyte biomarker, expression across the stratum basale has been previously described as a means of melanocyte quantification [22,23]. Immunofluorescence analysis reveals a uniform and regular distribution of melanocytes within untreated and low-fluence-exposed PHSEs (Figure 3D). However, when fluence was increased to moderate and high levels, melanocyte distribution decreased and became irregular. Furthermore, after exposure to both five consecutive low-fluence pulses and five chronic pulses delivered every 2–3 days across a two-week period, melanocyte distribution was significantly disrupted. Few melanocytes spaced irregularly across the stratum basale were observed following these more extreme treatments.

Quantification of melanocyte density across the tissue confirms these qualitative observations and demonstrates a dose-dependent decrease with increasing fluence for a single-pulse treatment (Figure 3E). A single low-fluence treatment resulted in a statistically significant decrease in melanocyte density, which decreased further with multiple-pulse treatments (Figure 3F).

Collectively, both the reduction in melanin content (which implies a reduction in melanin-rich cells) and reduction in melanocytes, which by definition are rich in melanin, suggest that the IPL treatments had the desired effect upon their target cells. However, to provide more direct evidence of these effects, we investigated the incidence of apoptotic changes at both the cellular and tissue levels.

**Figure 4:**
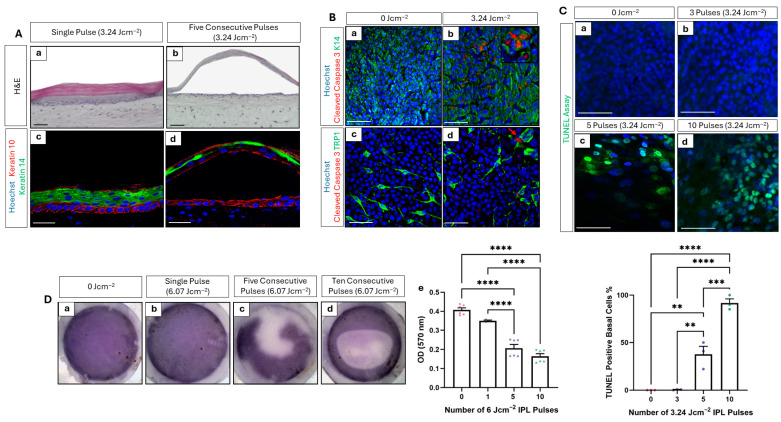
Apoptosis is induced in melanin-rich bioengineered tissue following IPL exposure. Representative haematoxylin and eosin (H&E) staining of PHSEs exposed to a single or five consecutive pulses of 3.24 Jcm^−2^ IPL and harvested 48 h after exposure (**Aa**,**b**). After a single pulse, tissue morphology remains unaffected, whereas following five consecutive pulses, the epidermis appears detached and blister-like. Immunofluorescence analysis of tissue cross-sections reveals disrupted expression of epidermal differentiation biomarkers (keratin 10—red, keratin 14—green, nuclei stained by Hoechst in blue) following five consecutive IPL pulses (**Ac**,**d**). Epidermal whole-mount immunofluorescence staining (**B**) reveals co-localisation of cleaved caspase 3 (red, arrow) and keratinocyte biomarker—keratin 14 (K14, green (**Ba**,**b**)) and melanocyte biomarker—TRP1 (green, (**Bc**,**d**)) in tissue constructs exposed to low-fluence IPL. TUNEL assay was used to fluorescently tag double-stranded DNA breaks in nuclei of apoptotic cells (green), with increased staining visible with increased number of consecutive low-fluence IPL emissions (**Ca**–**d**), the quantification of which revealed a statistically significant increase with the number of consecutive 3.24 Jcm^−2^ treatments (**Ce**) (data represent mean ± SEM, n = 3 representative of measurements taken from five images per sample obtained from three technical replicates from three independently constructed PHSEs). In all cases, nuclei are counterstained by Hoechst in blue. PHSEs 48 h post-exposure to multiple (1–10) high-fluence IPL pulses were incubated with the metabolic dye, MTT. Gross appearance of each tissue construct (**Da**–**d**) reveals significant zones of inhibition following five (**Dc**) and ten pulse treatments (**Dd**). Optical density (OD) of MTT precipitate production was quantified (**De**) to confirm a statistical reduction with increasing number of IPL pulses (data represent mean ± SEM, n = 3 representative of three measurements taken from three independently constructed PHSEs). Scale Bars: 50 μm, ** = *p* < 0.01, *** = *p* < 0.001, **** = *p* < 0.0001.

Cross-sectional histological analysis of PHSEs exposed to a single low-fluence IPL treatment (Figure 4(Aa)) revealed an organised, multicellular, stratified epidermis with expected cellular morphology and expected expression of the epidermal biomarkers keratin 14 and keratin 10 within the stratum basale and suprabasal layers, respectively (Figure 4(Ac)). However, histological analysis of PHSEs exposed to five consecutive low-fluence pulses resulted in the disorganisation of the tissue architecture. The epidermis following IPL exposure had detached from the underlying tissue and formed a blister-like structure (Figure 4(Ab)). Immunofluorescence analysis revealed the presence of keratin 14-positive cells lining the dermal compartment, with very little keratin 10 expression in the detached area of the epidermis (Figure 4(Ad)). This pronounced disruption of the tissue architecture reflects apoptosis-induced alterations at the tissue level.

Within the epidermis of the PHSE, there are two populations of cells, keratinocytes and melanocytes, both of which contain melanin. Given the changes in melanocyte density and pigmentation, it was important to identify the specific cell population(s) impacted by the IPL treatment. For this reason, epidermal whole-mount dual-immunostaining was conducted to ascertain which cell types within the stratum basale expressed cleaved caspase 3 and therefore reached critical temperature in response to IPL treatment. PHSEs were exposed to a low-fluence IPL treatment and harvested 48 h post-exposure. Co-localisation of cleaved caspase 3 and keratin 14 (K14), a biomarker specific for basal keratinocytes, was identifiable in IPL-treated PHSEs (Figure 4Bb, red arrow), but was notably absent from control samples (Figure 4(Ba)). Similarly, co-localisation of cleaved caspase 3 and the melanocyte specific biomarker TRP1 was also evident in IPL-treated PHSEs only (Figure 4(Bd), red arrow). Together, these data demonstrate that both melanin-rich cellular populations within the epidermis are impacted by IPL and undergo heat shock-induced apoptosis.

Furthermore, TUNEL staining, a widely adopted assay that detects dsDNA breaks consistent with apoptosis, was also used to further evidence apoptotic activity. Whole-mount epidermal staining depicts increasing TUNEL positive nuclei with an increasing number of low-fluence IPL pulses (Figure 4C). No identifiable TUNEL staining was observed in the control samples (Figure 4(Ca)) or samples that received three consecutive pulses of low-fluence IPL treatment (Figure 4(Cb)). In contrast, TUNEL-positive nuclei emerged in samples exposed to five consecutive low-fluence pulses (Figure 4(Cc)), and almost all nuclei appear TUNEL-positive following ten consecutive low-fluence pulses (Figure 4(Cd)). This was confirmed through quantification of TUNEL-positive cells (Figure 4(Ce)). The percentage of positive cells significantly increased following a five-pulse treatment and reached close to 100% following a ten-pulse treatment.

The use of metabolic dyes as a tool to measure the localised photothermal impact of laser treatments has been described in the literature [35,36,37]. In this study, we have adapted the well-established MTT assay for this purpose, which is a cell viability test routinely practised in toxicological and cytotoxicity testing based upon the conversion of MTT reagent into purple formazan crystals by metabolically active cells. PHSEs exposed to multiple consecutive high-fluence (6.07 Jcm^−2^) IPL treatments were returned to culture for 48 h to provide time for cells to respond to the stimulus. Following this incubation, an MTT assay was performed.

PHSEs that were not exposed to any IPL pulse appear homogeneous and uniformly purple, which indicates the presence of metabolically active cells distributed across the tissue construct (Figure 4(Da)). Similarly, PHSEs exposed to a singe high-fluence pulse also appear uniform in colouration (Figure 4(Db)). However, following five consecutive pulses, a clear zone of inhibition is detectable, which appears white in contrast to the purple precipitate within the surrounding viable cells (Figure 4(Dc)). Furthermore, after ten consecutive IPL pulses, a clear zone of inhibition within the centre of the PHSE is visible (Figure 4(Dd)). These data provide information regarding the spatial impact of IPL on the tissue, with a stark zone of inhibition reminiscent of the microscopic treatment zone (MTZ), a histological hallmark of laser treatments. Quantification of MTT precipitate retrieved from lysed PHSEs reveals a decrease in OD in a dose-dependent manner with an increasing number of high-fluence IPL pulses (Figure 4(De)). This reduction is statistically significant after both five and ten consecutive high-fluence pulses, supporting the visual evidence of MTT distribution across the PHSE.

Collectively, these data provide an abundance of both direct and indirect evidence as to the induction of apoptosis by IPL treatments. They also suggest that multiple pulses are required in order to achieve significant apoptosis in this tissue, supported by significant changes in TUNEL-positive cell number and cell viability assessments. This supports in vivo consumer experience, as multiple, frequent, treatment regimes are often required upon initiation of treatment.

### 3.4. IPL-Associated Inflammatory Response

The targeted destruction of cells by high-fluence clinical IPL delivery has been associated with the release of pro-inflammatory factors that can induce hyperpigmentation in consumers of darker skin tones [13], and, for this reason, IPL is contraindicated in darker skin tones. Pigmentary changes are primarily associated with high-fluence clinical treatments, rather than the relatively low fluence delivered by HUDs. To explore this aspect of IPL-mediated biological activity within keratinocytes and melanocytes, we measured several parameters consistent with the inflammatory response in vitro using a HUD with deactivated safety features to allow for in vitro delivery.

**Figure 5:**
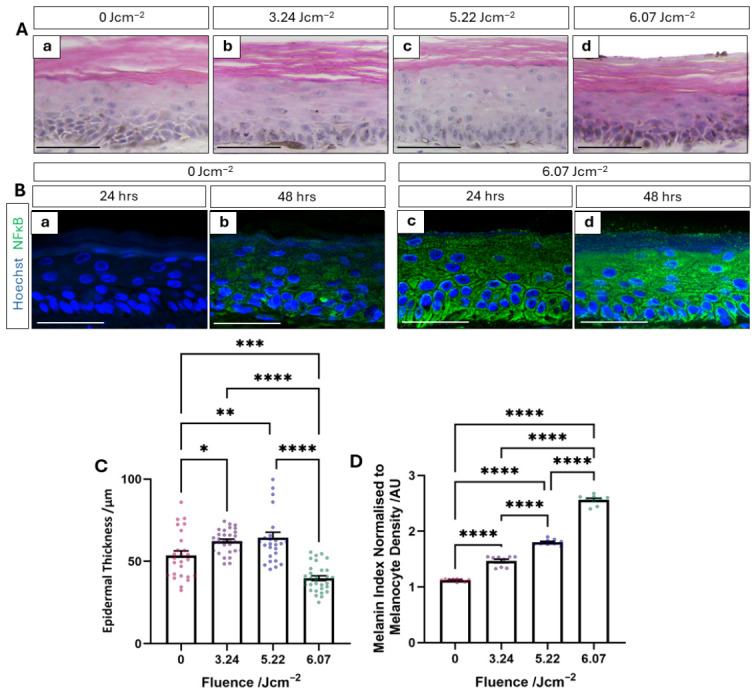
High-fluence IPL treatment gives rise to tissue hallmarks associated with inflammation. Haematoxylin and eosin (H&E) staining of PHSEs exposed to IPL treatments of increasing fluence and harvested for analysis 48 h post-treatment (**Aa**–**d**) reveal adequate tissue morphology, with some stark differences in epidermal thickness being apparent. Immunofluorescence staining of the transcription factor, NFκB ((**B**), green), responsible for the activation of inflammation-associated gene expression, reveals heightened expression at both 24 (**Bc**) and 48 h (**Bd**) post-high fluence IPL treatment compared with the time-matched control (**Ba**,**b**). Nuclei were stained by Hoechst in blue. Quantification of epidermal thickness (**C**) reveals a significant increase at low (3.24 Jcm^−2^) and moderate (5.22 Jcm^−2^) fluence, whilst high-fluence treatment (6.07 Jcm^−2^) significantly reduces epidermal thickness (data represent mean ± SEM, n = 3, representative of five measurements obtained per image, from three images per construct and three independently constructed PHSEs). The melanin index was normalised to melanocyte density (**D**) in order to assess the melanogenic potential of the tissue following IPL treatment, which significantly increased, correlating with increased fluence (data represent mean ± SEM, n = 3 representative of normalised data sets obtained from three measurements per construct of three independently constructed PHSEs). Scale Bars: 50 μm, * = *p* < 0.05, ** = *p* < 0.01, *** = *p* < 0.001, **** = *p* < 0.0001.

In our previous study, we demonstrated the relationship between epidermal thickness and exposure to an inflammatory stimulus [27]. Histological evaluation of PHSEs exposed to low, moderate, and high fluence also exhibit changes in epidermal thickness that correlate with the degree of IPL energy experienced by the tissue construct (Figure 5A). Most notably, the epidermis of those exposed to moderate fluence appears thicker, and those exposed to high fluence appears thinner than their control counterparts. This observation is confirmed by quantification of epidermal thickness, which demonstrates a statistically significant increase at both low and moderate fluence (Figure 5C). Interestingly, epidermal thickness also significantly decreased with high-fluence treatment. This is consistent with previous findings in that epidermal thickness increased when exposed to a mild inflammatory insult and decreased when exposed to a more extreme inflammatory stimulus within the context of tape strip removal [27]. Therefore, the changes in epidermal thickness observed post-IPL exposure are indicative of the tissue constructs’ expected response to inflammation.

In order to better explore any pro-inflammatory changes in the tissue post-IPL, we examined the expression of the transcription factor NFĸB. NFĸB is responsible for the regulation of genes involved in the pro-inflammatory response such as cytokines and cell proliferation-associated proteins [38]. NFĸB expression is increased at 24 h post-high-fluence IPL treatment compared with the time-matched control, in which staining is not visible (Figure 5B). Similarly, 48 h post-IPL exposure, staining intensity increased with high-level expression visible throughout the whole epidermis, and although positive staining is visible in the time-matched control, it remains significantly reduced compared with the IPL-treated sample. This upregulation supports the notion that PHSEs respond in an a pro-inflammatory manner when exposed to high-fluence IPL.

Interestingly, the quantification of skin tone indicates an increase in melanin index following a single, high-fluence IPL treatment (Figure 3B), despite a significant reduction in melanin-producing melanocytes (Figure 3E). Given that post-inflammatory hyperpigmentation is a consumer tension associated with IPL treatment, this observation has the potential to provide physiologically relevant insights into the consumer experience in vitro. To explore this phenomenon in greater depth, we normalised the melanin index to melanocyte density, which produced a notable fluence-dependent trend (Figure 5D). Even PHSEs exposed to low-fluence treatments exhibited a statistically significant increase in this metric, which further increased in-line with fluence. This indirectly suggests that, although fewer melanocytes remain in PHSEs exposed to IPL, they potentially display enhanced melanogenesis, most likely a consequence of pro-inflammatory factor release. However, despite demonstrating a fluence-dependent relationship, this is an indirect suggestive measurement, and further quantitative methods for expressing the key components implicated in the melanogenesis pathway are required to confirm this observation.

**Figure 6:**
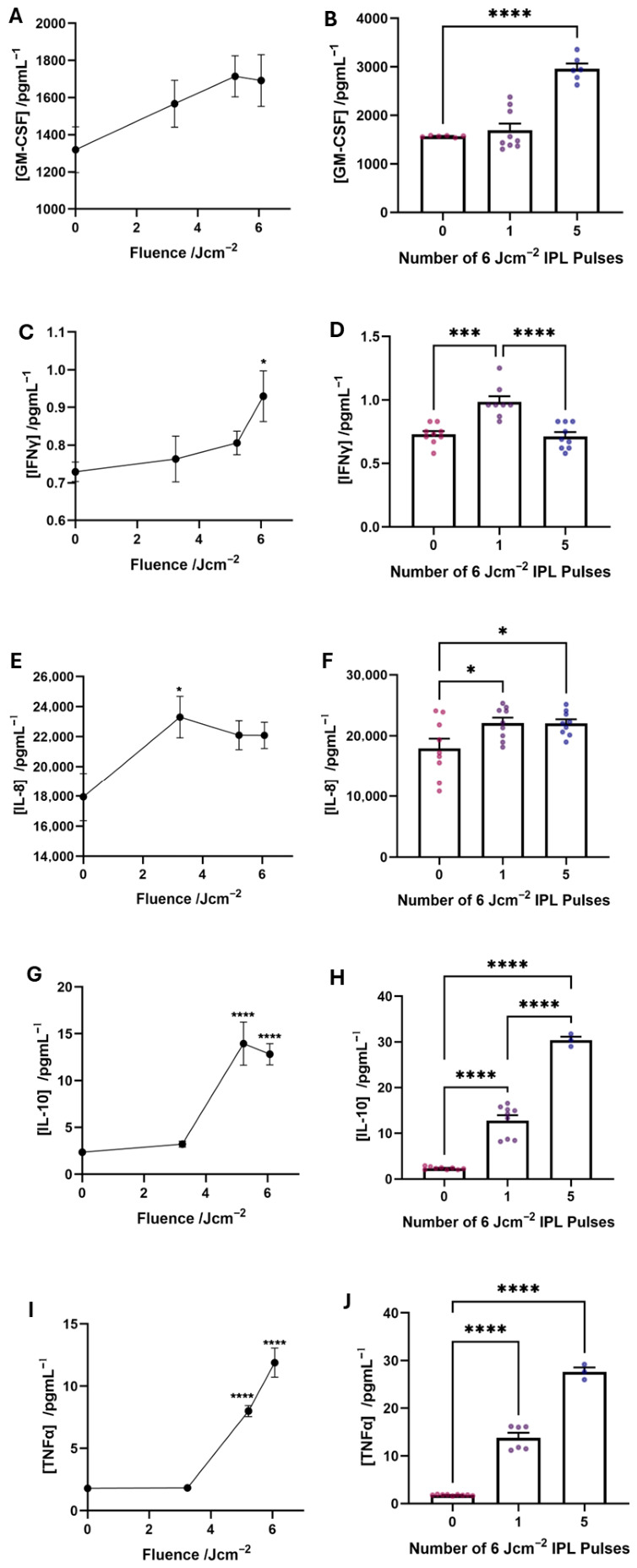
Increased secretion of cytokines and pro-inflammatory mediators from PHSEs exposed to IPL treatments. The cytokine content of culture medium obtained from PHSEs 48 h post-IPL exposure was analysed by multiplex analysis. The concentrations of GM-CSF (**A**,**B**), IFNγ (**C**,**D**), IL-8 (**E**,**F**), IL-10 (**G**,**H**) and TNFα (**I**,**J**) within the culture medium increases both with increasing fluence and the number of consecutive, high-fluence pulses delivered to the tissue construct (data represent mean ± SEM, n = 3, representative of duplicate measurements obtained from culture medium of 3 PHSEs per condition per independent experiment, of which there were 3). * = *p* < 0.05, *** = *p* < 0.001, **** = *p* < 0.0001.

To better understand the dynamics of inflammatory mediators within the system, a cytokine array was conducted on a conditioned cell culture medium harvested from cultures 48 h post-IPL treatment, many of which correlate with IPL fluence and the number of high-fluence consecutive pulses. The release of GM-CSF, a cytokine involved in the recruitment of immune cells in response to an inflammatory stimulus [39], increased in a dose-dependent manner with increasing fluence (Figure 6A). Expression also increased with the number of high-fluence pulses, reaching statistical significance after five consecutive pulses (Figure 6B). Although little has been reported to-date on the response of GM-CSF to IPL treatments, its release has been linked to the heat shock response initiated by heat shock protein upregulation [39,40,41]. As we have documented increased heat shock protein expression post-IPL, it is logical to conclude that GM-CSF expression was likely stimulated by this mechanism.

Similarly, IFNγ is a cytokine released by keratinocytes in response to stress [42]. There is evidence in the literature that IFNγ release is associated with the upregulation of cleaved caspase 3 and can induce apoptosis specifically in melanocytes, as it is thought to be a mediator involved in the pathogenesis of vitiligo, a skin condition characterised by the autoimmune destruction of melanocytes [43,44,45]. The release of IFNγ increases with fluence reaching a significantly elevated level with high-fluence treatment (Figure 6C), and a single high-fluence pulse resulted in the largest increase in secretion compared with the delivery of five consecutive pulses (Figure 6D).

Likewise, IL-8 is also a pro-inflammatory factor involved in the onset of hyperpigmentary changes [46], the release of which is associated with hsp70 expression [47]. IL-8 secretion is also significantly increased with increasing fluence (Figure 6E) and the number of high fluence pulses delivered (Figure 6F). Therefore, upregulation of both IL-8 and IFNγ may provide some mechanistic understanding and potential intervention strategies capable of modulating the post-IPL hyperpigmentary response.

On the other hand, IL-10 is an anti-inflammatory cytokine released by stressed tissues to modulate inflammation and resolve tissue damage [48]. The release of IL-10 was also fluence-dependent (Figure 6G), increasing significantly at moderate and high fluence, along with an increased number of high-fluence pulses (Figure 6H). This suggests that the tissue construct attempts to restore homeostasis and dampen the heightening inflammatory response. Interestingly, an increase in IL-10 has been documented in an immortalised keratinocyte line when exposed to 4–7 Jcm^−2^ IPL in 2D culture, a finding that corroborates our data set [46].

Finally, TNFα, a potent and universal pro-inflammatory mediator directly upregulated in response to NFκB expressions [49], showed a stark increase in expression following moderate- and high-fluence IPL exposure (Figure 6I). Similarly, a significant increase in TNFα release was observed following a single pulse of high-fluence IPL and five consecutive high-fluence pulses (Figure 6J). This dose-dependent relationship was expected, given that TNFα has been linked to the exposure of cells to temperatures in excess of 40 °C and exogenous the application of heat shock proteins [50,51]. TNFα is also one of the major contributors involved in the onset of post-inflammatory hyperpigmentation [52].

Together, these data help us to build an understanding of the biological mechanisms that underpin the response of melanin-rich tissues to IPL through the assessment of cellular and molecular changes in response to IPL-mediated temperature changes.

## 4. Discussion

Intense Pulsed Light (IPL) is a method of photoepilation that harnesses the absorptive spectrum of melanin, a natural chromophore, resulting in targeted thermolysis of cells in the bulb region of the hair follicle and, ultimately, hair follicle shedding [12]. The ability of IPL to specifically target melanin-rich cells for thermolysis lends itself to a wide array of cosmetic and dermatological applications in addition to hair removal, including treatment of pigmented lesions [53,54], signs of photoageing, vascular lesions [55,56], and skin texture improvement [57,58,59]. In addition, but IPL therapy has also been described in the improvement of vascular remodelling [60,61], anti-inflammatory responses [62], enhanced collagen synthesis [63,64], and as a treatment for dry eye disease and meibomian gland dysfunction within the field of ophthalmology [65,66]. Due to the variety and quantity of these emerging applications, there is a growing parallel need for the development of robust in vitro techniques capable of modelling the biological impact of IPL at a cellular and molecular level within the context of distinct tissue types.

The adoption of home use devices (HUDs) that allow consumers to experience salon-quality photoepilation treatments within their own home has driven an increase in the popularity of IPL as a means of epilation. As technology advances and treatments become more efficacious, possessing the ability to screen treatment regimes and obtain fundamental insights into the cellular pathways involved has become more critical. Current approaches aimed at investigating the hair removal capability of IPL treatments rely heavily on clinical trials [14,15,17]. Unfortunately, clinical trials are both time-consuming and expensive, often relying on the collection of skin biopsies for in-depth molecular analyses that are both invasive and are require specialised clinical staff. For this reason, in vitro approaches offer an alternative screening platform capable of providing pre-clinical, predictive data to streamline the clinical trial process. However, there are limited published approaches that either utilise 2D culture techniques or isolated hair follicles [11,18], both of which are far removed from the in vivo complex multi-cellular environment, which in turn limits their predictive capacity.

In this study, we present a novel, innovative approach utilising a melanin-rich PHSE to model the thermodynamic response of melanin-rich cells to IPL. This pioneering application has provided evidence that cells within the construct not only respond to IPL in a fluence or dose-dependent manner, but also undergo each stage of thermal shock, leading to apoptosis, consistent with the cellular changes that occur around the hair follicle in vivo, which is pivotal to hair removal [1]. Expression of hsp70 and cleaved caspase 3, biomarkers consistent with the cellular heat shock response, increased both in response to increasing fluence and the number of pulses delivered, demonstrating clear dose-dependent trends. This evidence is consistent with increased cellular temperature, induced through exposure of the tissue construct to IPL energy [67,68,69]. In vivo, increased cellular temperature results in selective apoptosis, leading to hair follicle shedding and desired hair removal [69]. We have successfully measured a range of metrics consistent with increased cellular apoptosis, including reduced tissue pigmentation, a reduction in melanocyte density, histological hallmarks consistent with apoptosis, and TUNEL assay positivity. Collectively, this strong body of evidence demonstrates the robust response of the PHSE to IPL in a manner consistent with expected biological action.

Unlike widely adopted approaches in the literature that utilise ex vivo biopsies as a model system, engineered tissue constructs provide an almost unlimited sample source capable of high-throughput and destructive analysis and that can provide more in-depth analyses. Here, we demonstrate a large array of analytical approaches, including traditional histological techniques, immunofluorescence detection of biomarkers, secretion of pro-inflammatory factors, and metabolic dye conversion. This litany of analytical approaches demonstrate the flexibility and breadth of application of in vitro approaches, with the ability to provide robust pre-clinical data of predictive value whilst maintaining consumer relevance.

Interestingly, many of the metrics used to investigate the biological action of IPL throughout this study demonstrate increased expression following multiple or chronic IPL exposure. This is consistent with in vivo reports that multiple IPL sessions are required upon commencement of a hair removal regime to achieve maximum efficacy [70]. We also provide a biological rationale as to why this is the case, as the expression of key pathway components such as cleaved caspase 3, which is indicative of the threshold cellular temperature being reached, increased significantly with the number of pulses. This provides validation that our model system reflects the in vivo biological process, but also that this system lends itself to chronic studies, representative of consumer regimes.

Furthermore, expression of a range of pro-inflammatory cytokine mediators, along with histological changes consistent with tissue inflammation and expression of the transcription factor NFĸB, correlate with the increasing intensity of IPL treatments. Here, we offer mechanistic insights into this phenomenon, demonstrating an increase in the melanogenic activity of remaining melanocytes following IPL treatment, which coincides with an increase in the key cytokines responsible for the onset of post-inflammatory hyperpigmentation. Further supporting evidence from the literature has documented an increase in GM-CSF and IL-10 post-IPL [48] whilst also providing novel insights into the expression of other cytokines such as IFNγ, IL-8, and TNFα with increasing fluence and multiple treatments. The ability to model this downstream response to IPL provides a useful tool that is capable of providing consumer relevant insights, not only into the efficacy of treatments, but also into the tensions associated with high-fluence treatments. This methodology, therefore, has the capability to screen interventions capable of dampening the pro-inflammatory response, such as topical formulations pre- or post-treatment, along with providing fundamental insights into the incidence of such tensions across specific consumer demographics.

## 5. Conclusions

In this study, we demonstrate the unique and pioneering application of a melanin-rich, bioengineered tissue construct as a platform technology capable of probing the cellular and molecular events that unfold in response to IPL. This innovative study tracks the cellular response from IPL energy delivery, inferring increased cellular temperature, and ultimately thermolysis, and the key cellular events that govern IPL-mediated photoepilation. Although the model system presented in this study lacks the hair follicle appendage and therefore heat conduction via the hair shaft, important information regarding the intracellular processes initiated by IPL-exposure has been presented. In addition, we also demonstrate the increased expression of key pro-inflammatory mediators following more extreme IPL treatments, supporting our mechanistic understanding of post-inflammatory hyperpigmentation in vitro. However, such extreme treatment regimens are not consistent with correct HUD use, and the safety features that prevent the over-delivery of high fluence were disabled for this study. Therefore, these findings support the necessity of such safety features and the contraindication of IPL use in consumers with dark skin tones, whilst demonstrating cellular response to extreme IPL exposure. By modelling these cellular events in vitro, we provide a platform that is capable of generating pre-clinical predictive data along with screening topical formulations and efficacy comparisons.

## Data Availability

The original contributions presented in this study are included in the article/Supplementary Material. Further inquiries can be directed to the corresponding author.

## Supplementary Materials

The following supporting information can be downloaded at https://www.mdpi.com/article/10.3390/cells15100859/s1. Table S1: Primary Antibodies used in Immunofluorescence Staining.

## Abbreviations

The following abbreviations are used in this manuscript:

HUD	Home Use Device
IPL	Intense Pulsed Light
PHSE	Pigmented Skin Equivalent
PBS	Phosphate-Buffered Saline
NCS	Neonatal Calf Serum
FM	Fontana–Masson
H&E	Hematoxylin and Eosin
MI	Melanin Index
ALI	Air-liquid Interface
ECM	Extracellular Matrix
