# Supplementary material for: An Innovative Bioengineering Approach to Investigate the Response of Melanin-Rich Cells to Intense Pulsed Light (IPL)
Source: Cells. 2026 May 8;15(10):859. doi: 10.3390/cells15100859 (PMC13204098; doi:10.3390/cells15100859)
Supplement: Supplementary file 1 [file cells-15-00859-s001.zip › cells-4243890-supplementary.pdf]

Target Antigen	Supplier	Product Code	Dilution
Hsp70	Abcam	ab2787	1:100
Cleaved Caspase 3	Cell Signalling Technology	9661	1:100
TRP1	Abcam	ab190709	1:100
NFkB	Abcam	ab32360	1:100
Cytokeratin 10 (K10)	Abcam	ab76318	1:1,000
Cytokeratin 14 (K14)	Abcam	ab7800	1:1,000

Supplementary Table S1: Primary Antibodies used in Immunofluorescence Staining
Table detailing the primary antibodies used in immunofluorescence staining of tissue constructs, including their supplier, product code and working dilution.
